# First Outbreak of NDM-1-Producing *Klebsiella pneumoniae* ST11 in a Portuguese Hospital Centre during the COVID-19 Pandemic

**DOI:** 10.3390/microorganisms10020251

**Published:** 2022-01-23

**Authors:** Gabriel Mendes, João F. Ramalho, Aida Duarte, Adriana Pedrosa, Ana Cristina Silva, Lucía Méndez, Cátia Caneiras

**Affiliations:** 1 Microbiology Research Laboratory on Environmental Health (EnviHealthMicro Lab), Institute of Environmental Health (ISAMB), Faculty of Medicine, Universidade de Lisboa (ULisboa), 1649-026 Lisboa, Portugal; gabriel-mendes@campus.ul.pt (G.M.); jfrancisko.ramalho@gmail.com (J.F.R.); lucia.gonzalez@chedv.min-saude.pt (L.M.); 2 Faculty of Pharmacy, Universidade de Lisboa (ULisboa), 1649-033 Lisboa, Portugal; aduarte@ff.ulisboa.pt; 3 Egas Moniz Interdisciplinary Research Center, Egas Moniz University Institute, 2829-511 Monte da Caparica, Portugal; 4 Microbiology Laboratory, Clinical Pathology Department, Centro Hospitalar entre Douro e Vouga, 4520-211 Santa Maria da Feira, Portugal; adriana.pedrosa@chedv.min-saude.pt (A.P.); anac.silva@chedv.min-saude.pt (A.C.S.); 5 Pulmonology Department, Centro Hospitalar entre Douro e Vouga, 4520-221 Santa Maria da Feira, Portugal; 6 Institute of Preventive Medicine and Public Health (IMP&SP), Faculty of Medicine, Universidade de Lisboa (ULisboa), 1649-026 Lisboa, Portugal

**Keywords:** *Klebsiella pneumoniae*, *E. coli*, outbreak, ST11, KL105, NDM-1, whole genome sequencing, carbapenemase, virulence, hospital environment, Portugal

## Abstract

New Delhi metallo-β-lactamase (NDM) carbapenemase has been considered a global threat due to its worldwide widespread in recent years. In Portugal, a very low number of infections with NDM-producing Enterobacterales has been reported. A total of 52 strains from 40 patients and 1 environmental sample isolated during COVID-19 pandemic were included in this study. Wholegenome sequencing (WGS) was performed on 20 carbapenemase-producing strains, including 17 NDM-1-producing *Klebsiella pneumoniae* ST11-KL105 lineage strains, one NDM-1-producing *Escherichia coli* ST58 strain and one KPC-3-producing *K. pneumoniae* ST147 strain, recovered from a total of 19 patients. Of interest, also one NDM-1-producing *K. pneumoniae* ST11-KL105 was collected from the hospital environment. Genome-wide phylogenetic analysis revealed an ongoing dissemination of NDM-1-producing *K. pneumoniae* ST11 strains (*n* = 18) with the same genetic features seen across multiple wards. Furthermore, the ST58 *E. coli* strain, collected from a patient rectal swab that was also colonised with a *K. pneumoniae* strain, also showed the IncFIA plasmid replicon and the *bla*_NDM-1_ gene (preceded by IS30 and followed by genes *ble*_MBL_, *trpF*, *dsbC*, *cutA*, *groES* and *groEL*). The *bla*_NDM-1_ is part of Tn*125-like* identical to those reported in Poland, Italy and India. The *bla*_KPC-3_ *K. pneumoniae* ST147-KL64 strain has the genetic environment Tn*4401d* isoform. In conclusion, herein we report the molecular epidemiology, resistome, virulome and mobilome of the first NDM-1 carbapenemase outbreak caused by *K. pneumoniae* ST11-KL105 lineage during the COVID-19 pandemic in Portugal. Moreover, the outbreak strains characterised included seventeen different patients (infected and colonised) and one environmental sample which also emphasises the role of commensal and hospital environment strains in the dissemination of the outbreak.

## 1. Introduction

Infections caused by carbapenemase-producing Enterobacterales (CPE), are among the leading global threats to public health, where the prevalence of multidrug-resistant forms have increased substantially in recent years [1]. In fact, a large number of carbapenemases have been increasingly identified worldwide in *Klebsiella pneumoniae*, placing it as one of the top international threats [2]. Among these emerging resistance genes, New Delhi metallo-β-lactamase (NDM) has been considered a major challenge due to its ability to hydrolyse a wide range of β-lactams and its rapid spread [3]. NDM carbapenemases have been mainly detected in *K. pneumoniae* and *Escherichia coli* and to a lesser extent from other bacterial species [4]. NDM-1 was first identified in a *K. pneumoniae* strain from a Swedish patient that travelled to New Delhi in 2008 [5]. Epidemiological studies indicate that intercontinental travel to endemic areas, such as the Indian subcontinent and the Middle East, promotes the worldwide spread of clinical strains harbouring the *bla*_NDM-1_ gene [3,6].

Indeed, the presence of NDM-1 in *K. pneumoniae* has already been reported in several European countries [7]. In particular, recent NDM-1 outbreak reports have been published in Spain [8,9,10]. In Portugal, one of Spain’s neighbouring countries, the number of NDM-1 producing Enterobacterales strains detected until now has been low, with only sporadic cases reported, but none of them in *K. pneumoniae* [11,12,13]. Consequently, the aim of this study is to report and characterise an NDM-1 producing *K. pneumoniae* hospital outbreak in Portugal.

## 2. Materials and Methods

### 2.1. Hospital Setting and Outbreak Description

The outbreak was first noted in August 2020 in a *K. pneumoniae* strain, during a regular infection control screening. The recently implemented carbapenemase immunochromatographic detection method by the Microbiology Laboratory, Clinical Pathology Service, led to the identification of consecutive carbapenemase-producing *bla*_NDM_-producing Enterobacterales strains. Considering that NDM is not a usual carbapenemase found in this hospital and in Portugal, the Microbiology Laboratory and the Program for Prevention and Control of Infection and Antimicrobial Resistance (PPCIRA) team have activated the epidemiological surveillance mechanisms for detection of carbapenemase-producing bacteria through cultural tests and/or polymerase chain reaction (PCR) and subsequent sequencing of strains recovered from infection or colonisation cases. Additional searches were performed on the same ward, on the hospital environment, and on different medical and surgical wards (located at different floors of the hospital). Since the first *bla*_NDM_-positive strain identified, additional infection prevention and control measures have been immediately established, such as additional cleaning and disinfection reinforcement and an active surveillance of individuals at risk, namely individuals who were in close contact with identified patients. A close contact is defined by Centers for Disease Control and Prevention (CDC) as someone who was within 2 m of an infected person for at least 15 min within a 24-h period starting from 2 days before illness onset (or, for colonisation or asymptomatic cases 2 days prior to positive specimen collection) until the time the patient is isolated (https://www.cdc.gov, accessed on 28 December 2021); hospitalised in services with identified patients or in high-risk services such as intensive care units (ICU). Moreover, rectal swabs of patients upon entry to the hospital and between wards transfer were performed. All positive cases were further put into contact isolation. Between August 2020 and December 2020, 40 patients were included in the study due to development of infection or colonisation following hospital admission. Infection was defined by clinical and laboratory criteria, while colonisation was defined by the absence of relevant clinical symptoms. Effective communication between the Microbiology Laboratory and the elements of PPCIRA (Program for the Prevention and Control of Infections and Antimicrobial Resistance) was extremely important for the rapid and effective establishment of measures to control the outbreak.

### 2.2. Bacterial Strains

Fifty-one strains from 40 patients plus 1 environmental sample were included in this study, for a total of 52 strains. The samples were collected from an Hospital Centre in northern Portugal, August–December 2020, using standard clinical operating procedures. Identification was performed by microbiology laboratories using conventional methods or automated systems such as MicroScan WalkAway (Beckman Coulter, Lisboa, Portugal)). Rectal swabs were seeded in a chromogenic culture medium CHROMagar™ mSuperCARBA™ (CHROMagar, Frilabo, Portugal). For further molecular characterisation, all strains were maintained frozen at −80 °C in BHI broth (VWR Prolabo, Lisboa, Portugal) plus 15% glycerol. For analysis purposes, the strains were grown overnight in BHI Broth (18 h at 37 °C) and seeded on Mueller-Hinton agar (VWR, Lisboa, Portugal).

### 2.3. Antimicrobial Susceptibility Testing

Antibiotic susceptibility test was performed using the standardised Kirby–Bauer disk diffusion technique, in accordance with the European Committee on Antimicrobial Susceptibility Testing (EUCAST) guidelines. The detailed methodology is available at http://www.eucast.org/ast_of_bacteria/disk_diffusion_methodology/, accessed on 28 December 2021. Detailed instructions for Mueller–Hinton agar medium (VWR Prolabo^®^, Lisboa, Portugal), including preparation and storage, are also available in the same EUCAST guidelines document.

Susceptibility was tested for several antibiotics: amoxicillin/clavulanic acid (20/10 µg), cefoxitin (30 µg), cefotaxime (5 µg), ceftazidime (10 µg), imipenem (10 µg), gentamicin (10 µg), ciprofloxacin (5 µg), tigecycline (15 µg), aztreonam (30 µg), ertapenem (10 µg), meropenem (10 µg), doripenem (10 µg) and ceftazidime/avibactam (10/4 µg) (Biorad, Lisboa, Portugal). The strains were categorised as susceptible, standard dosing regimen (S); susceptible, increased exposure (I); and resistant (R) by applying the breakpoints in the phenotypic test results. A complementary Etest^®^ (BioMérieux, Marcy l’Étoile, France) for ceftazidime/avibactam was also performed. The inhibition zones were interpreted according to the EUCAST breakpoints (version 11.0, 2021) (available at https://eucast.org/clinical_breakpoints/, accessed on 28 December 2021). Multidrug-resistant (MDR) bacteria were defined as those that acquired non-susceptibility to at least one agent in three or more antimicrobial categories, in accordance with the United States Centre for Disease Control and Prevention (CDC) and the European Centre for Disease Prevention and Control (ECDC) consensual definition [14].

### 2.4. Molecular Methods and Detection of Carbapenemase Genes

A search for carbapenemases genes was first performed at the hospital with the immunochromatographic detection methods RESIST-3 O.K.N (Coris BioConcept. Belgium) or GeneXpert Carba-R assay (Cepheid, Frankfurt, Germany) and afterwards confirmed by polymerase chain reaction (PCR). PCR-based screening was performed to identify carbapenemases genes with primers designed for *bla*_OXA-48_ F:5′-GGCTGTGTTTTTGGTGGCATC-3′; R:5′-GCAGCCCTAAACCATCCGATG-3′, *bla*_KPC_ [15], *bla*_VIM_ [16], *bla*_NDM_ [5], *bla*_GES_ [17], *bla*_CTX-M-15_ [18]), using the NZYTaq II 2x Green Master Mix (NZYTech, Lisboa, Portugal) following the manufacturer’s instructions. The PCR products were resolved in 1% agarose gel in 10Xconcentrated Tris-Borate-EDTA (TBE buffer) (NZYTech, Lisboa, Portugal). The PCR assays included positive and negative controls. The positive controls were previously purified using the ExoCleanUp FAST (VWR Prolabo^®^, Lisboa, Portugal) kit and sequenced at STABVida Portugal, as well as after purification the positive amplified carbapenemase genes.

The BLAST program (available at the National Centre for Biotechnology Information website (http://www.ncbi.nim.nih.gov/, accessed on 28 December 2021) was used to search for nucleotide sequences. Multiple-sequence alignments were performed with the Clustal Omega program, available at (https://cge.cbs.dtu.dk/services, accessed on 28 December 2021).

### 2.5. Whole Genome Sequencing (WGS)

The collection used for whole genome sequencing (WGS) analysis represent a total of 20 nonduplicate strains, based on their phenotypic and genotypic resistance determinants previously accessed by antimicrobial susceptibility test and PCR-based screening for carbapenemases genes, respectively. These strains belonged to 19 patients and one strain was recovered from environmental sample.

The genomic DNA was extracted for WGS from cultures grown overnight in Mueller–Hinton agar, using the NZY Tissue gDNA Isolation kit (NZYTech, Lisboa, Portugal), as per the manufacturer’s recommendations and sent to STABVida Portugal for sequencing. Indexed libraries were prepared using the KAPA HyperPrep Library Preparation Kit (Roche, Switzerland), according to the manufacturer’s recommended protocol and the sequence was performed using an Illumina HiSeq Novaseq 6000 platform with paired-end reads (2 X151 bp).

The raw data quality control was performed using FASTQC v0.11.9, and the trimming and de novo assembly was performed using CLC Genomics Workbench 12.0.3 (QIAGEN, Aarhus, Denmark). All assemblies were carried out with automatic word size, similarity fraction of 0.95, a length fraction of 0.95 and a minimum contig size of 500 bp. In silico core genome multilocus sequence typing (cgMLST) and the single-nucleotide polymorphism (SNP) analysis were performed using a bacterial whole genome sequence typing and source tracking database (BacWGSTdb) [19,20,21]. The genomes of 21 bacterial strains were compared against the reference genome of a *K. pneumoniae* ST11 strain, HS11286 (NCBI Reference sequence: CP003200) and the derived SNP data were used for further phylogenetic analysis. One strain collected from the outbreak event (FMUL433) was used to help root the phylogenetic tree. The tree was depicted using the Interactive Tree of Life tool version 6.5 (iTOL, https://itol.embl.de/, accessed on 28 December 2021) [22].

### 2.6. Drug Resistance Associated Genes, Virulence Genes, Capsular Types and Plasmid Replicons

Antimicrobial resistance genes, virulence genes, capsular type (K-locus) and O-locus types as well as the Multilocus Sequence Typing (MLST) were identified using a *K. pneumoniae*-specific genomic typing tool (Kleborate), available at https://github.com/katholt/Kleborate, accessed on 28 December 2021. Plasmid analyses were identified using the PlasmidFinder database (https://cge.cbs.dtu.dk/services/PlasmidFinder/, accessed on 28 December 2021) (cut-off values: minimum of 60% coverage and 95% identity) and using the BacWGSTdb server (available at http://bacdb.cn/BacWGSTdb/, accessed on 28 December 2021). For virulence factors analysis, the VFDB tool was used (http://www.mgc.ac.cn/VFs/, accessed on 28 December 2021).

### 2.7. Ethical Approval

The study was approved by Centro Hospitalar de Entre o Douro e Vouga Ethics Committee (Nr. CA-330/2020-0t_MP/AC). Strains were obtained as part of routine diagnostic testing, and were analysed anonymously to respect patient privacy. The epidemiological data were obtained retrospectively from clinical records and the study proposal was analysed and dismissed from evaluation by the Ethics Committee of the Lisbon Academic Medical Centre of the Faculty of Medicine, University of Lisbon, Portugal (Nr. 248/21).

## 3. Results

### 3.1. Outbreak Description of Clinical Strains

A total of 52 samples retrieved from 40 patients and 1 environmental sample were collected between August–December 2020, in a hospital Centre in northern Portugal. Out of the 52 samples, the great majority were *K. pneumoniae* strains (51/52; 98.1%), with one strain belonging to the *E. coli* species (1/52; 1.9%). Regarding the environmental sample collected (*n* = 1), the strain was *K. pneumoniae*. According to Table 1, among the 40 patients, 70% (28/40) were male and 30% female (12/40). The average age observed was 71.2 years (range from 38 to 94). These patients were admitted in distinct hospital wards: Internal medicine (*n* = 17), Surgery (*n* = 6), Neurology (*n* = 5), Emergency (*n* = 4), Intensive Care Unit (ICU) (*n* = 3), Urology (*n* = 2), Pulmonology (*n* = 2), Orthopaedics (*n* = 2), Cardiology (*n* = 1) and Acute Care (*n* = 1). Three patients stayed in more than one ward (P15, P20, P30) as identified in Table 1. Fourteen out of 51 human samples (27.5%) were obtained from 3 different clinical specimens: urine (*n* = 10), ascitic fluid (*n* = 3) and blood (*n* = 1), with P15 had simultaneously both urine and blood strains. The remaining 37 samples (72.5%) were collected from rectal swabs, and two rectal swabs from three patients each (P7, P9, P32) were collected.

### 3.2. Antimicrobial Susceptibility

The antimicrobial susceptibility test was performed for all 52 strains and uniformly showed resistance to ciprofloxacin, gentamicin, ceftazidime cefotaxime, cefoxitin, imipenem, meropenem, ertapenem, doripenem, aztreonam and amoxicillin/clavulanic acid (52/52; 100%). Fifty strains showed resistance to ceftazidime/avibactam (50/52; 96.2%) and 9 strains showed resistance to tigecycline (9/52; 17.3%).

### 3.3. Identification of Carbapenemase Genes

PCR amplification of carbapenemase genes was performed in all 52 strains, with 49 strains harbouring the *bla*_NDM-1_ gene (49/52; 94.2%) and 1 isolate harbouring the *bla*_KPC-3_ gene (1/52; 1.9%). No carbapenemase genes were detected in the two remaining strains (2/52; 3.8%).

### 3.4. WGS Analysis

WGS was conducted on 20 strains, namely 19 human samples from 19 different patients (18 *K. pneumoniae* strains and 1 *E. coli* strain) and 1 environmental sample (*K. pneumoniae*) with the objective of understanding the evolutionary epidemiology, resistome, virulome and mobilome of some of the presumed outbreak cases. Eighteen *K. pneumoniae* strains belonged to sequence type 11 (ST11), included in worldwide disseminated clonal group 258 (CG258) and the remaining *K. pneumoniae* strain belonged to ST147. Interestingly, the environmental strain (FMUL 403) was among the 18 *K. pneumoniae* ST11 strains. Furthermore, the *E. coli* strain belonged to ST58.

Table 2 shows the genetic features of 20 WGS strains. All *K. pneumoniae* ST11 strains produced the *bla*_NDM-1_ carbapenemase gene, as well as the CTX-M-15, SHV-11, OXA-1 and TEM-1 beta-lactamases. Furthermore, all harboured the *aac*(3)-*IId, aac*(6′)-*Ib*-*cr, strA* and *strB* aminoglycoside resistance genes. The resistance to quinolones were conferred by chromosomal mutations in *gyrA* (A83I) and *parC* (C80I) loci, as well as by plasmid-encoded *qnrB*1 gene. Moreover, all strains had resistance genes to tetracycline (*tetD*), sulphonamide (*sul*2) and trimethoprim (*dfrA14*). Moreover, one strain (FMUL386; P36) had the *catB4* phenicol resistance gene. The environmental *K. pneumoniae* ST11 had the same genetic features as the other strains collected from 17 patients.

Two strains were identified from the colonisation samples producing carbapenemases. The *K. pneumoniae* ST147 contained the genes that code for KPC-3 carbapenemase, SHV-11 beta-lactamase and presented one mutation in both *gyrA* (A83I) and *parC* (C80I) loci. The second strain was an *E. coli* ST58 co-producing NDM-1 with several beta-lactamases such as CTX-M-15, AmpC1, OXA-1 and TEM-1. Furthermore, other antibiotic resistance genes were identified conferring aminoglycoside (*aac*(3)-*IId, aac*(6′)-*Ib*-*cr, aadA, strA* and *strB*) and quinolone resistance (*qnrB1* and *qnrS1*). Moreover, genes that confer resistance to other antibiotics were also found such as macrolide (*mphB*), phenicol (*cmlA1*), sulphonamide (*sul2* and *sul3*), tetracycline (*tetA* and *tetD*) and the trimethoprim (*dfrA14* and *dfrA15*).

Moreover, regarding the virulence genes identified in *K. pneumoniae* outbreak strains, including the environmental sample, the antigen O encoded on *rfb* locus type O2v2 and the polysaccharide capsule encoded in K-loci (KL105) were identified. The *rcsA* and *rcsB* genes responsible for regulation of capsule synthesis were found, however the genes regulator of mucoid phenotype A (*rmpA* and *rmpA2*) were absent. Other important virulence factors were identified, fimbria adhesins type 1 (*fimA-fimK* genes) and type 3 (*mrkA-mrkJ* genes); the iron uptake systems: enterobactin cluster *(entA- fes* genes), aerobactin (*iutA*), salmochelin (*iroE* and *iroN*), yersiniabactin cluster (*fyuA-ybtX* genes), which was included in integrative conjugative element (ICEKp10). All strains showed the same plasmid replicons: IncFIA(HI1), IncFIB(K) and IncR.

Relatively to colonisation strains, *K. pneumoniae* ST147 and *E. coli* ST58 both producing carbapenemases of Class A and B have different genetic features. The *K. pneumoniae* ST147 had the capsular KL64 and antigen O2v1 loci and the same virulence factors (fimbria and iron uptake genes) as of *K. pneumoniae* ST11-KL64 strains, except an ICEKp12 harbouring the ybt 16 gene. Moreover, it carried three IncFIB(pKPHS1), IncFII(K), IncN plasmid replicons. The *E. coli* ST58 showed a higher number of the resistance genes together with virulence genes. Two out of five plasmid replicon IncFIA(HI1) and IncR were found in outbreak strains. Of relevance, BLAST analysis of the contigs carried the replicon IncFIA(HI1) and IncR of *E. coli* ST58 compared to the draft genome of NDM-1-producing *K. pneumoniae* (2 from patients and one environmental strain) that showed a range of 99.3 to 99.5% match with IncFIA(HI1), and not showing similitude with plasmid replicon IncR.

The genetic environments of *bla*_NDM-1_ and *bla*_KPC-3_ genes on *K. pneumoniae* were also searched. The *bla*_NDM-1_ gene, in both *K. pneumoniae* ST11-KL105 and *E. coli* ST58 strains, was preceded by a complete copy of insertion sequence IS30 and followed by genes *ble*_MBL_ (conferring resistance to bleomycin), *trpF*, *dsbC*, *cutA*, *groES* and *groEL*. The *bla*_NDM-1_ is part of Tn*125-like* which is 100% identical to *K. pneumoniae* reference sequences from Poland (Genbank MW363916.1), Italy (Genbank MK467522) and India (Genbank CP030858). The *bla*_KPC-3_ *K. pneumoniae* ST147-KL64 strain has the genetic environment Tn*4401d* isoform, which is characterised by a 68 bp deletion between *istB* and the *bla*_KPC_ gene.

**Figure 1:**
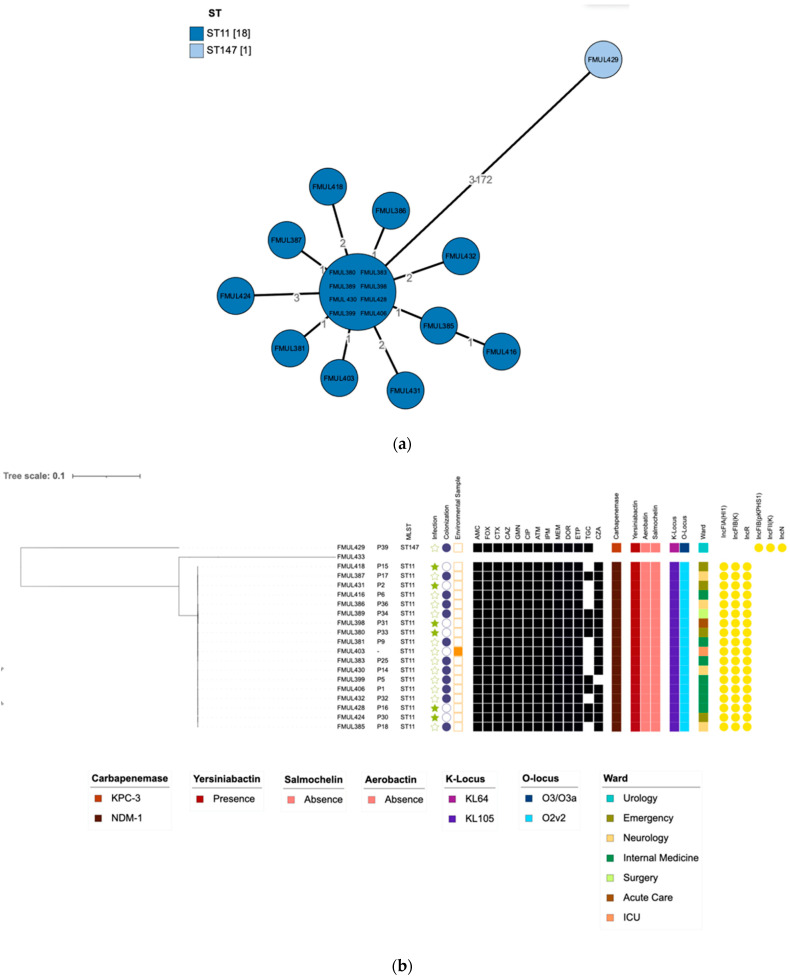
(**a**) Phylogenetic relationship between the *K. pneumoniae* clinical strains by core genome multilocus sequence typing (cgMLST) analysis, visualised in a minimum-spanning tree, showing distance based on cgMLST genes. (**b**) Neighbour-joining tree based on SNPs calling for *K. pneumoniae* clinical strains showing the relationship between strains. The tree was rooted with the help of FMUL433 strain sequence recovered from the outbreak event. The tree was annotated with phenotypic drug susceptibility data to ciprofloxacin (CIP), ceftazidime (CAZ), imipenem (IPM), cefotaxime (CTX), cefoxitin (FOX), gentamicin (GMN), amoxicillin/clavulanic acid (AMC), aztreonam (ATM), ceftazidime/avibactam (CZA), tigecycline (TGC), ertapenem (ETP), meropenem (MEM) and doripenem (DOR), where black squares indicate phenotypic resistance (R). Coloured strips from left to right indicate the carbapenemase produced, yersiniabactin, salmochelin and aerobactin presence/absence, capsular locus type (KL), antigen O locus type (OL) and ward of isolation of the strains are also annotated on the tree. Plasmid replicons are displayed in yellow circles.

The genome assemblies of the 20 sequenced strains were also analysed by comparison to the sequences deposited in the BacWGSTdb server. The allelic distances were visualised in a minimum-spanning tree. Furthermore, a neighbour-joining tree was generated using the whole genome SNPs approach, using the *K. pneumoniae* ST11 strain, HS11286 (NCBI Reference sequence: CP003200) as a reference genome. The results by these two complementary genome-wide approaches (Figure 1) showed that all ST11 strains were genetically close to each other (<4 cgMLST loci differences). As expected, ST147 *K. pneumoniae*, were not genetically related to the ST11 K*. pneumoniae* strains, as they differ by over 100 SNPs or cgMLST loci [19].

Overall, the data obtained both by SNP and cgMLST analysis resulted in the grouping of 18 NDM-1-producing *K. pneumoniae* ST11 strains that may be responsible for the spread across multiple wards in this hospital Centre. These 18 strains had very similar phenotypic resistance, harbouring the same resistance and virulence genes, with the same capsular locus (KL105), antigen locus (O2v2) and carrying the same plasmid replicons.

## 4. Discussion

Dissemination of *K. pneumoniae* strains harbouring the carbapenemase NDM-1 continues to increase, with various outbreak reports worldwide, including in several European countries in recent years [23,24,25,26,27,28,29,30]. Regarding the recent molecular studies on carbapenem-producers performed in Portugal [31,32,33,34,35], only a few reports of NDM-positive Enterobacterales strains were described [11,12,13]. Of relevance, none of them being in *K. pneumoniae* strains, indicating the limited presence of this metallo-beta-lactamase gene in the country.

The data gathered from this study highlight the increasing problem of multidrug resistance, all 52 samples displayed either carbapenemase (NDM-1 in 49 strains and KPC-3 in 1 isolate) or ESBL genes (CTX-M-15). In fact, all strains were resistant to most antibiotics tested, as usually found in NDM-1 positive strains [36]. However, 41 NDM-1 and 1 KPC-3 strains remain susceptible to tigecycline.

WGS analysis by both cgMLST and SNP approach revealed that 18 out of the 21 analysed strains linked to the outbreak event were very closely related, as shown by their lower number of differences (<4 cgMLST loci). These 18 samples were all NDM-1-producing *K. pneumoniae* strains belonging to ST11. As expected, *K. pneumoniae* ST147 strain did not belong to the outbreak event since they differ from over 100 SNPs or cgMLST loci, as previously reported [19]. The outbreak occurred in multiple wards, including in the operating room where an environmental sample was collected, further stressing the importance of the environment in the dissemination of the strain causing the outbreak. These results highlight the dissemination of the NDM-1-producing *K. pneumoniae* strains in the hospital centre. It also emphasises the importance of the fast implementation of additional infection control measures in order to promote an efficient and rapid detection of colonisation/infection and decontamination of different environmental sources across the hospital wards.

The ST11 *K. pneumoniae* strains belong to CG258, which is responsible for most of carbapenem-resistant *K. pneumoniae* infections in Europe [37]. Although a previous report showed a molecular epidemiology change in the last few years in Portugal, in which non–clonal group 258 high-risk clones among *K. pneumoniae* carbapenemase producers have started to emerge [38], the findings in the present study demonstrate that the dissemination of CG258, namely ST11, is still occurring, particularly in the northern part of the country. According to Bonnin et al. [38] the emergence and dissemination of non-CG258 KPC-Kp isolates in France was linked to dissemination of these clones from Portugal, which can be explained by the high migratory flow of Portuguese from the northern region of Portugal to France. Moreover, the authors state that KPC-Kp epidemiology has changed in Europe, at least in France and Portugal, where CG258 is not the most prevalent clone.

Furthermore, of interest, the outbreak strains described herein had the same capsular loci KL105 and antigen O2v2 serotype. This *K. pneumoniae* ST11-KL105 lineage was previously described in northern Portugal, in non-producing carbapenemase strains [39], as well as in China [40,41] in a strain harbouring both KPC-2 and NDM-1 enzymes collected from a patient with bloodstream infection [40]. The Asian region, particularly China, has been affected with several outbreaks with a large variety of clones that mainly express NDM-1 and OXA-48-like carbapenemases, especially through the dissemination of the ST11 clone, the most prevalent ST among carbapenem resistant *K. pneumoniae* in the country [42]. Although no clear link or background information regarding any connections with China was available for this study, the increase in migratory flow into Portugal for the last 10 years indicates that the number of Asian residents in Portugal have increased considerably (Data available at https://www.pordata.pt/Home, accessed on 2 November 2021). However, more studies are required in order to establish a clear relationship between these migratory flows.

All 19 *K. pneumoniae* strains harboured the IncFIB(pKPHS1), IncFII(K), IncR plasmid replicons. Among the several plasmids characterised in *K. pneumoniae*, the IncFIIK and IncFIBK are considered the most prevalent across this species [37]. Furthermore, the *bla*_NDM_ genes mostly carry conjugative plasmids belonging to several incompatibility types, including IncFIA, IncFIB, IncN, IncR, IncFII, IncHI1, IncF, IncP, IncY, IncFIIA, IncI [8,9,43,44,45]. We highlighted significant epidemiological equality either the phenotype or genotype of *K. pneumoniae* lineage (ST11-K105) and plasmids IncFIA(HI1); IncFIB(K); IncR disseminated worldwide.

The *bla*_NDM-1_ gene was also present in one *E. coli* strain collected from a patient during the outbreak event that was also colonised by an NDM-1-producing *K. pneumoniae*. This strain belongs to ST58 (clonal complex ST155), a globally disseminated clone previously reported in humans, animals and in the environment (Enterobase 2021, available at https://enterobase.warwick.ac.uk/species/index/ecoli, accessed on 31 October 2021). It also harbours the CTX-M-15 enzyme, and contains several other resistance genes, including genes for AmpC enzymes. Moreover, it had a virulence score of 4, based on the presence of the genes encoding for yersiniabactin, aerobactin and salmochelin [46]. The concomitant high prevalence of iron siderophores genes and MDR genes is worrisome and requires further attention, suggests its great potential for hypervirulence and pathogenicity.

It is interesting that the NDM-producing *K. pneumoniae* outbreak has occurred during the coronavirus disease 2019 (COVID-19) pandemic, whilst several Infection Prevention and Control (IPC) measures have been adopted to reduce nosocomial microorganism transmission. Bentivegna, E. et al. have reported that health care-associated infection (HCAI) caused by *C. difficile* incidence was significantly lower with respect to the previous years [47]. Indeed, a significant reduction in the incidence of total MDR bacterial infections was observed during the pandemic compared to in pre-pandemic years (*p* < 0.05) but, of relevance, the authors revealed a significantly higher incidence of MDR bacterial infections in COVID-19 departments compared with other medical departments [48], probably caused by the increase of empiric antimicrobial consumption [48,49]. Moreover, extended-spectrum β-lactamase (ESBL)-producing *K. pneumoniae* have been reported as the pathogen presenting the highest increase [48]. Molecular surveillance, infection control measures by rapid and efficient screening of patients as well as periodic environmental screening are essential for controlling and preventing the dissemination of highly pathogenic carbapenemase-producing strains in hospital setting.

## 5. Conclusions

In conclusion, we describe the molecular epidemiology, resistome, virulome and mobilome of the first hospital outbreak caused by NDM-1-producing *K. pneumoniae* ST11-KL105 lineage in Portugal during the COVID-19 pandemic, highlighting its high pathogenicity and easy dissemination. Herein, we also report an hypervirulent NDM-1-producing *E. coli* ST58 strain and emphasise the role of commensal and hospital environment *K. pneumoniae* strains in the dissemination of the outbreak.

## Figures and Tables

**Table 1:** Clinical features of all bacterial strains retrieved from patients and hospital environment.

Patient	Organism	Age (Gender)	Hospital Ward	Date of Isolation	Biological Product	Colonisation Site
P1	*K. pneumoniae*	47 (M)	Internal Medicine	18/09/2020	-	Rectal swab
P2	*K. pneumoniae*	55 (F)	Emergency	05/12/2020	Ascitic fluid	-
P3	*K. pneumoniae*	62 (M)	Surgery	29/08/2020	-	Rectal swab
P4	*K. pneumoniae*	64 (F)	Urology	28/09/2020	Urine	-
P5	*K. pneumoniae*	65 (M)	Internal Medicine	10/09/2020	-	Rectal swab
P6	*K. pneumoniae*	93 (M)	Internal Medicine	09/10/2020	-	Rectal swab
P7	*E. coli*	66 (M)	Internal Medicine	26/09/2020	-	Rectal swab
	*K. pneumoniae*			26/09/2020	-	Rectal swab
P8	*K. pneumoniae*	92 (F)	Internal Medicine	31/08/2020	-	Rectal swab
P9	*K. pneumoniae*	62 (M)	Internal Medicine	26/08/2020	-	Rectal swab
				07/10/2020		Rectal swab
P10	*K. pneumoniae*	94 (F)	Internal Medicine	03/10/2020	-	Rectal swab
P11	*K. pneumoniae*	38 (M)	ICU	19/09/2020	-	Rectal swab
P12	*K. pneumoniae*	85 (F)	Internal Medicine	26/08/2020	-	Rectal swab
				03/11/2020	Urine	-
P13	*K. pneumoniae*	79 (M)	Internal Medicine	01/10/2020	-	Rectal swab
P14	*K. pneumoniae*	59 (M)	Neurology	16/11/2020	-	Rectal swab
P15	*K. pneumoniae*	80 (M)	Internal Medicine	02/10/2020	-	Rectal swab
			Emergency	11/10/2020	Urine	-
			Emergency	11/10/2020	Blood	-
P16	*K. pneumoniae*	77 (M)	Internal Medicine	09/11/2020	Urine	-
P17	*K. pneumoniae*	56 (M)	Pulmonology	28/08/2020	-	Rectal swab
P18	*K. pneumoniae*	65 (M)	Neurology	27/08/2020	-	Rectal swab
P19	*K. pneumoniae*	87 (F)	Internal Medicine	12/10/2020	Urine	-
P20	*K. pneumoniae*	64 (M)	ICU	17/09/2020	-	Rectal swab
			Surgery	06/10/2020	Urine	-
P21	*K. pneumoniae*	81 (F)	Neurology	14/10/2020	-	Rectal swab
P22	*K. pneumoniae*	76 (F)	Cardiology	31/08/2020	-	Rectal swab
P23	*K. pneumoniae*	84 (M)	Orthopedics	29/08/2020	-	Rectal swab
P24	*K. pneumoniae*	79 (M)	Neurology	04/11/2020	Urine	-
P25	*K. pneumoniae*	61 (M)	Internal Medicine	26/08/2020	-	Rectal swab
P26	*K. pneumoniae*	81 (F)	Internal Medicine	15/10/2020	-	Rectal swab
P27	*K. pneumoniae*	83 (F)	Internal Medicine	28/10/2020	-	Rectal swab
P28	*K. pneumoniae*	55 (M)	Pulmonology	26/08/2020	-	Rectal swab
P29	*K. pneumoniae*	77 (M)	ICU	12/10/2020	-	Rectal swab
				12/10/2020	Ascitic fluid	-
P30	*K. pneumoniae*	89 (M)	Internal Medicine	30/08/2020	-	Rectal swab
			Emergency	26/10/2020	Urine	-
P31	*K. pneumoniae*	77 (M)	Acute Care	28/08/2020	-	Rectal swab
				01/09/2020	Urine	-
P32	*K. pneumoniae*	63 (F)	Internal Medicine	12/09/2020	-	Rectal swab
				15/12/2020		Rectal swab
P33	*K. pneumoniae*	56 (F)	Emergency	25/08/2020	Urine	-
P34	*K. pneumoniae*	81 (M)	Surgery	28/08/2020	-	Rectal swab
P35	*K. pneumoniae*	56 (M)	Surgery	13/09/2020	Ascitic fluid	-
				17/09/2020	-	Rectal swab
P36	*K. pneumoniae*	62 (M)	Neurology	27/08/2020	-	Rectal swab
P37	*K. pneumoniae*	81 (M)	Surgery	28/08/2020	-	Rectal swab
P38	*K. pneumoniae*	74 (M)	Surgery	29/08/2020	-	Rectal swab
P39	*K. pneumoniae*	59 (M)	Urology	11/11/2020	-	Rectal swab
P40	*K. pneumoniae*	83 (M)	Orthopedics	30/08/2020	-	Rectal swab
-	*K. pneumoniae*	-	Operation room *	14/09/2020	-	-

Legend: F, female; M, male; * collected from disinfection room drain.

**Table 2:** Genetic features (MLST, resistance and virulence profile, capsular and antigen loci and plasmid replicons of 20 multidrug-resistant strains obtained from 19 infected/colonised patients and 1 environmental samples.

Species	MLST	Patients	Environmental Sample	Resistance Profile (Number of Strains)				Virulence Profile		Capsular Locus (KL) Antigen Locus (OL)		Plasmid Replicons
				*bla_*Carb	β-Lactams	Other Resistance Genes	Fimbriae	ICEKp	Iron Uptake	K_Locus	O_Locus	
*Klebsiella pneumoniae*	ST11	17	1	NDM-1 (*n* = 18)	CTX-M-15; SHV-11; OXA-1; TEM-1 (*n* = 18)	*aac(3)-IId; aac(6′)-Ib-cr; strA; strB; qnrB1; tet(D); sul2; dfrA14; gyrA*-83I*; parC-*80I *(n = 18); catB4* (*n* = 1)	*fimA-fimK; mrkA-mrkJ*	*ybt* 10; ICE*Kp*4 (*n* = 18)	enterobactin (*entA-fes*), aerobactin (*iucA-iutA*), salmochelin (*iroE-iroN*), yersiniabactin(*fyuA-ybtX*)	KL105 (*n* = 18)	O2v2 (*n* = 18)	IncFIA(HI1); IncFIB(K); IncR (*n* = 18)
*Klebsiella pneumoniae*	ST147	1	-	KPC-3	SHV-11	*gyrA*-83I; *parC*-80I; *fosA*	*fimA-fimK; mrkA-mrkJ*	*ybt* 16; ICE*Kp*12	enterobactin (*entA-fes*), aerobactin (*iutA*), salmochelin *(iroE-iroN*), yersiniabactin (*fyuA-ybtX*)	KL64	O2v1	IncFIB(pKPHS1);IncFII(K);IncN
*Escherichia coli*	ST58	1	-	NDM-1	CTX-M-15; AmpC1; OXA-1; TEM-1	*aac(3)-IId; aac(6’)-Ib-cr; aadA; strA; strB; qnrB1; qnrS1; mphB; cmlA1; sul2; sul3; tet(A); tet(D); dfrA14*	*fimA*-*fimI cfaA*-*cfaE*, *ecpA*-*ecpG*	*-*	aerobactin (*iucA*-*iutA*); salmochelin (*iroB-iroN*) yersiniabactin (*fyuA*-*ybtX*), iron/manganese (*sitA*-*sitD*)	-	-	Col440II, IncFIA(HI1), IncFIB(AP001918), IncFIC(FII), IncI1, IncR, IncX4
